# Interactions of Apigenin and Safranal with the 5HT1A and 5HT2A Receptors and Behavioral Effects in Depression and Anxiety: A Molecular Docking, Lipid-Mediated Molecular Dynamics, and In Vivo Analysis

**DOI:** 10.3390/molecules27248658

**Published:** 2022-12-07

**Authors:** Faiq Amin, Mahmoud A. A. Ibrahim, Syed Rizwan-ul-Hasan, Saima Khaliq, Gamal A. Gabr, Muhammad, Asra Khan, Peter A. Sidhom, Prashant Tikmani, Ahmed M. Shawky, Saara Ahmad, Syed Hani Abidi

**Affiliations:** 1 Department of Biological and Biomedical Sciences, Aga Khan University, Karachi 74800, Pakistan; 2 Computational Chemistry Laboratory, Chemistry Department, Faculty of Science, Minia University, Minia 61519, Egypt; 3 School of Health Sciences, University of KwaZulu-Natal, Westville, Durban 4000, South Africa; 4 Department of Computer Science, DHA Suffa University, Karachi 75500, Pakistan; 5 Department of Biochemistry, Federal Urdu University of Arts, Science and Technology, Karachi 75300, Pakistan; 6 Department of Pharmacology and Toxicology, College of Pharmacy, Prince Sattam Bin Abdulaziz University, Al-Kharj 11942, Saudi Arabia; 7 Department of Pharmaceutical Chemistry, Faculty of Pharmacy, Tanta University, Tanta 31527, Egypt; 8 Science and Technology Unit (STU), Umm Al-Qura University, Makkah 21955, Saudi Arabia; 9 Department of Biomedical Sciences, Nazarbayev University School of Medicine, Nur-Sultan 010000, Kazakhstan

**Keywords:** depression, anxiety, natural compounds, molecular docking and dynamics, serotonin receptors, murine model

## Abstract

Background: The current study utilizes in silico molecular docking/molecular dynamics to evaluate the binding affinity of apigenin and safranal with 5HT1AR/5HT2AR, followed by assessment of in vivo effects of these compounds on depressive and anxious behavior. Methods: The docking between apigenin and safranal and the 5HT1A and 5HT2A receptors was performed utilizing AutoDock Vina software, while MD and protein-lipid molecular dynamics simulations were executed by AMBER16 software. For in vivo analysis, healthy control (HC), disease control (DC), fluoxetine-, and apigenin-safranal-treated rats were tested for changes in depression and anxiety using the forced swim test (FST) and the elevated plus-maze test (EPMT), respectively. Results: The binding affinity estimations identified the superior interacting capacity of apigenin over safranal for 5HT1A/5HT2A receptors over 200 ns MD simulations. Both compounds exhibit oral bioavailability and absorbance. In the rodent model, there was a significant increase in the overall mobility time in the FST, while in the EPMT, there was a decrease in latency and an increase in the number of entries for the treated and HC rats compared with the DC rats, suggesting a reduction in depressive/anxiety symptoms after treatment. Conclusions: Our analyses suggest apigenin and safranal as prospective medication options to treat depression and anxiety.

## 1. Introduction

According to the World Health Organization (WHO, Geneva, Switzerland), depression is the main cause of disabilities worldwide, with an estimated 300 million people suffering from this disease globally [1]. It is classified as a mood disorder by the American Psychiatric Association Diagnostic and Statistical Manual of Mental Disorders (DSM-5) [2]. Depression disturbs patients’ emotions, cognition, and behavior, where low mood is the primary symptom [2].

The most widely used drugs are selective serotonin reuptake inhibitors (SSRIs) such as sertraline, fluoxetine, and paroxetine [3]. These drugs target the reabsorption of serotonin selectively and are effective for major depressive and anxiety disorders as well as, in high doses, for obsessive-compulsive disorder [3].

In the 7 subtypes of serotonin receptors, there are a total of 15 different receptors, which makes the role of serotonin in major depressive disorder (MDD) multifaceted and complicated [4]. Both the 5HT1A (inhibitory) and 5HT2A (excitatory) subtypes of serotonin receptors are known to have involvement in many important medical conditions [5]. They are most heavily concentrated and distributed widely in the human brain [6], with extensive evidence linking them with psychiatric ailments, including both depression and anxiety [7].

It has been noted that newer agents such as vilazodone and vortioxetine, which work as agonists at the 5HT1A receptor (5HT1AR), are effective in treating depression [8,9,10,11]. Additionally, atypical antipsychotics have a therapeutic effect, decreasing the symptoms of depression in treatment-resistant depression [12,13,14]. For example, olanzapine, an atypical antipsychotic, usually works on the 5HT2ARs by inhibiting them [15,16]. Both of these targets are similarly included in the antianxiety impacts of medicines that affect the serotonergic system, where 5HT1AR agonists and 5HT2AR antagonists function as anxiolytics [17,18].

Due to the presence of treatment-resistant depression (TRD) [19,20], there is an increased interest in alternative therapeutic options. The term treatment-resistant depression (TRD) represents what was previously labeled as dysthymia and chronic major depression, according to the DSM-5 (fifth edition; APA, 2013) [21]. These patients must present a depressed mood for a minimum of 2 years, as indicated in the manual. Furthermore, a recent review indicates that a diagnosis of TRD requires at least two treatment failures with the use of a standard dose for a suitable duration [22]. A recent review presenting a summary of several studies showed saffron (*Crocus sativus* L.) to be more efficient compared with a placebo in alleviating depression and at the very least equal to the standard dosages of both fluoxetine and imipramine (tricyclic antidepressant and SSRI, respectively) [23]. Two of the active ingredients in saffron—namely crocin and safranal—have been proven to have antidepressant action [24]. Similarly, chamomile (*Matricaria chamomilla* L.) extract has been shown to exhibit antidepressant activity in addition to its well-established anxiolytic activity [25]. Yi et al. showed that apigenin (an active component of chamomile) reduced forced swim test (FST)-induced disturbances in serotonin, dopamine, and their respective metabolites [26]. Interestingly, anxiety is present in about 60% of patients with depression as a comorbidity and resembles the scenario of double depression that involves major depression superimposed on dysthymia [3]. Anxiety is more common when major depression includes persistent depressive disorder [27]. Therefore, validated rat models of depression, such as streptozotocin (STZ)-induced diabetic rats [28,29,30,31], also include a component of anxiety in them as diabetes mellitus (DM) and MDD or anxiety are linked by several pathways, including the tryptophan catabolism (TRYCATs) pathway [32,33,34], advanced glycation end products (AGEs) [35], brain-derived neurotrophic factor (BDNF), and nuclear factor kappa B (NFKB) [36,37,38]. It is important to mention that the murine models with diabetes-induced depression and anxiety have previously been described as effective models to study MDD and anxiety, especially as the diabetes can be easily induced, and the mood symptoms follow quickly thereafter [39].

There are no experimental or computational studies implicating the direct effects of apigenin and safranal on the serotonergic receptors that underlie MDD. Computational models help investigate the basic premise of proposing a mechanism of action, namely the presence of interaction between a ligand and a medically important receptor. This is faster for identifying a target receptor while being cost-effective in comparison with the trial-and-error methods used in vitro and in vivo [40]. Functionally, the combined effect of apigenin and safranal on depression and anxiety has not been tested using in vivo models. Therefore, the current study aims to employ molecular docking and molecular dynamics approaches to analyze the molecular interactions between safranal and apigenin and the 5HT1A and 5HT2A receptors, followed by an in vivo analysis of these compounds on depressive and anxious behavior. Each inhibitor-receptor complex was further embedded in a POPC membrane bilayer and subjected to MD simulations over 200 ns in order to confirm their stability. Since this was preliminary work on safranal and apigenin, we formed two arms to the study where we captured the essence of these compounds in behavioral testing for the quantification of depression and anxiety in rats and probed into the bioinformatics of these molecules in docking on the two receptors most implicated in the pathogenesis of depression and anxiety.

## 2. Results

### 2.1. Domain Architecture Analysis and In Silico Computations

Analysis of the 5HT2AR and 5HT1AR structures revealed the presence of seven transmembrane loops and an internal G-protein-binding internal loop characteristic of G-protein-coupled receptors (GPCRs) [41].

**Figure 1:**
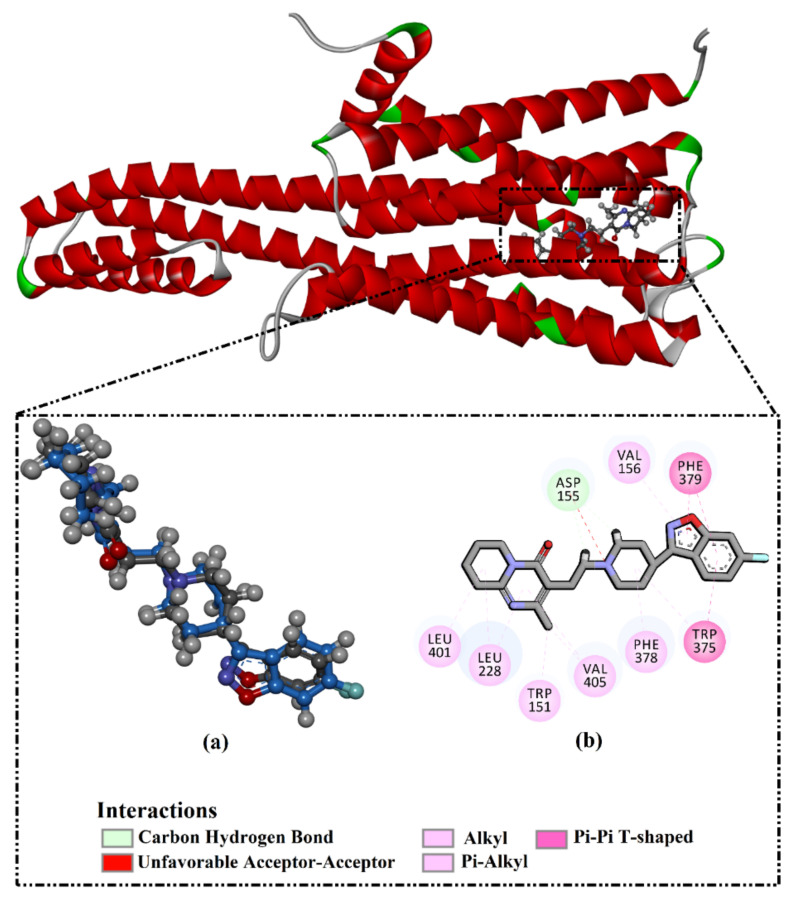
(**a**) A 3D representation of the native structure (gray) and portended docking pose (blue). (**b**) The 2D molecular interactions of the anticipated docking pose of 8NU ligand with 5HT2AR.

**Figure 2:**
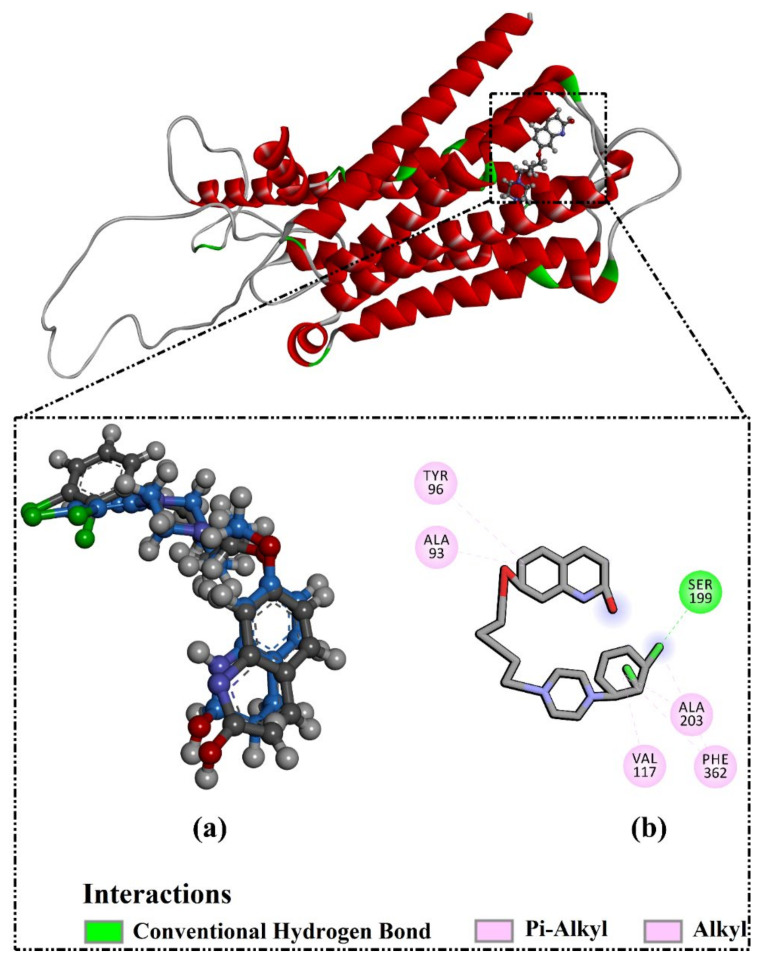
(**a**) A 3D representation of the native structure (gray) and portended docking pose (blue). (**b**) The 2D molecular interactions of the anticipated docking pose of 9SC ligand with 5HT1AR.

The validation of the performance of the AutoDock Vina1.1.2 (La Jolla, CA, USA)) software to predict the right binding mode of the 5HT2AR ligand was initially estimated. For validation, the co-crystallized 3-[2-[4-(6-fluoranyl-1,2-benzoxazol-3-yl)piperidin-1-yl]ethyl]-2-methyl-6,7,8,9-tetrahydropyrido [1,2-a]pyrimidin-4-one (8NU/risperidone) and 7-[4-[4-[2,3-bis(chloranyl)phenyl]piperazin-1-yl]butoxy]-3,4-dihydro-1H-quinolin-2-one (9SC/aripiprazole) ligands were redocked against the 5HT2AR and 5HT1AR, respectively, and the portended docking poses were compared to the co-crystallized binding modes (PDB codes: 6a93 [42] and 7e2z [43], respectively). The portended docking poses were almost identical to the native binding modes, with RMSDs of 0.25 Å and 0.33 Å and docking scores of −11.1 and −9.1 kcal/mol for the 5HT2AR and 5HT1AR, respectively (Figure 1 and Figure 2). Despite the inability of the 8NU ligand to form any hydrogen bonds inside the 5HT2AR binding pocket, other noncovalent interactions were noticed involving vdW, hydrophobic, and π-based interactions (Figure 1 and Figure 2). In conclusion, this data comparison revealed the perfect performance of AutoDock Vina software in anticipating the experimental 5HT2AR and 5HT1AR ligand poses.

**Figure 3:**
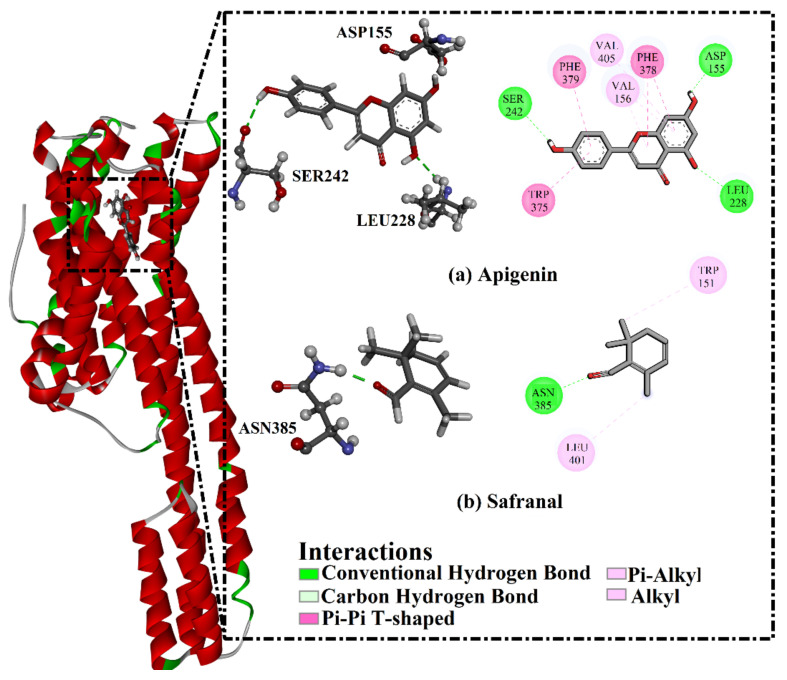
The predicted binding modes of (**a**) apigenin and (**b**) safranal with the 5HT2AR, in accordance with the last trajectory of the 200 ns MD simulations.

In the next step, apigenin and safranal were individually docked against the 5HT1AR and 5HT2AR. Based on the docking calculations, apigenin and safranal demonstrated docking scores of −8.9 and −6.8 kcal/mol against the 5HT2AR and −8.4 and −6.0 kcal/mol against the 5HT1AR, respectively. To achieve more reliable results, the predicted complexes were subjected to 200 ns molecular dynamics (MD) simulations. Based on the gathered trajectories over the MD simulations, the binding affinities were then estimated. Analysis of the ligand interactions with the 5HT2AR revealed that apigenin interacted with the 5HT2AR with a better binding affinity than safranal, with a Δ*G*_binding_ of −34.3 for apigenin and −17.1 kcal/mol for safranal (Table 1). Inspecting the binding mode of apigenin with the 5HT2AR demonstrated that the OH group of the phenol ring formed a hydrogen bond with the carbonyl group of SER242 with a bond length of 2.45 Å (Table 1 and Figure 3). Additionally, the two OH groups of resorcinol made two hydrogen bonds with the NH group of LEU228 and the COO group of ASP155 with bond lengths of 2.32 and 1.69 Å, respectively (Table 1 and Figure 3). Similarly, analysis of the binding mode of safranal inside the binding pocket of the 5HT2AR unveiled that the carbonyl group of cyclohexa-1,5-dienecarbaldehyde formed a hydrogen bond with the NH2 of ASN385 with a bond length of 1.90 Å (Table 1 and Figure 3).

**Figure 4:**
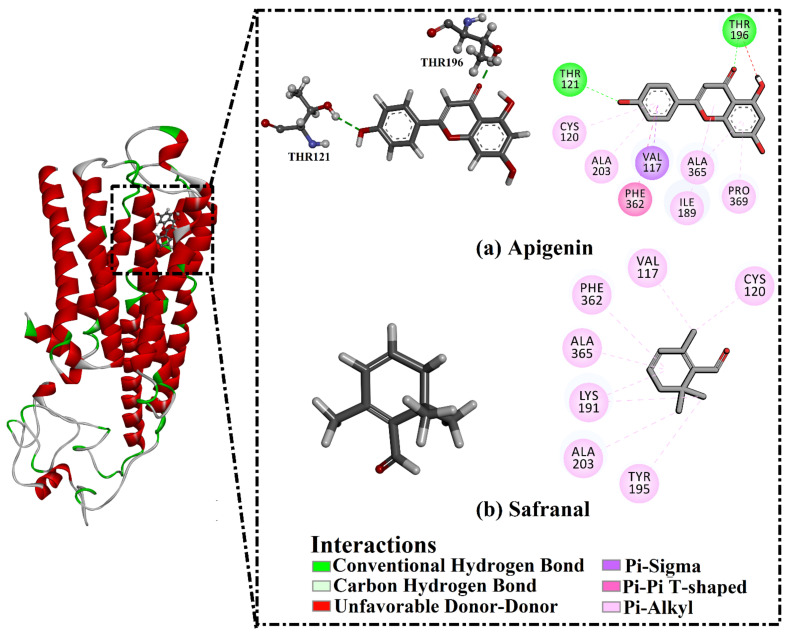
The predicted binding modes of (**a**) apigenin and (**b**) safranal with the 5HT1AR, in accordance with the last trajectory of the 200 ns MD simulations.

For the interaction of ligands with the 5HT1AR, apigenin and safranal displayed MM-GBSA binding energies of −26.8 and −22.3 kcal/mol, respectively (Table 1). Analysis of the binding mode of apigenin with 5HT1AR demonstrated that the oxygen of the phenol ring formed a hydrogen bond with the OH of THR121 with a bond length of 2.57 Å (Figure 4 and Table 1). Additionally, the oxygen of the 2,3-dihydropyran-4-one ring formed a hydrogen bond with the OH of THR196 with a bond length of 1.88 Å (Figure 4 and Table 1). In contrast, safranal could not form any hydrogen bonds with the key amino acids in the active site of the 5HT1AR (Figure 4 and Table 1).

### 2.2. Analysis of Binding Affinity Partitioning

**Figure 5:**
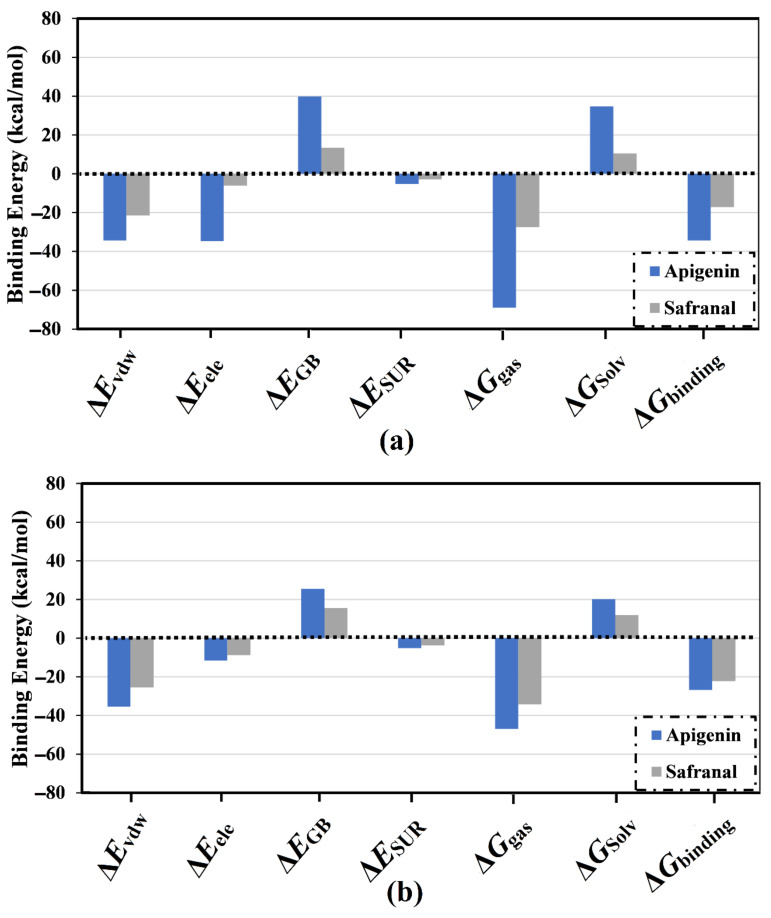
Participation of diverse energy components in the total MM-GBSA binding energies of apigenin and safranal complexed with (**a**) 5HT2A and (**b**) 5HT1A receptors.

The MM-GBSA scheme identifies various dissociation energy components that share the total binding energy (Δ*G*_binding_). These components include the total gas-phase energy (Δ*G*as), generalized Born equation (Δ*E*_GB_), van der Waals interactions (Δ*E*_vdw_), the nonpolar component of the solvation energy (Δ*E*_SUR_), solvation-free energy (Δ*G*_Solv_), and electrostatic interactions (Δ*E*_ele_). The calculated energy components for apigenin and safranal complexed with the 5HT2AR and 5HT1AR are listed in Figure 5a,b, respectively. Analysis of the dissociation energy components highlighted the considerable participation of the Δ*E*_vdw_ and Δ*E*_ele_ interactions in the stability of the drug-receptor binding affinities (Figure 5). Numerically, the van der Waals interactions (Δ*E*_vdw_) were found to be the predominant force involving molecular complexation with the 5HT2AR and 5HT1AR for both apigenin (Δ*E*_vdw_ of −34.7 and −35.4 kcal/mol, respectively) and safranal (Δ*E*_vdw_ of −21.5 and −25.5 kcal/mol, respectively). Similarly, electrostatic interactions (*E*_ele_) were also a prominent force involved in the interaction of apigenin and safranal complexed with the 5HT2AR and 5HT1AR, with binding affinities of −34.3 and −11.6 as well as −6.0 and −8.8 kcal/mol, respectively. It is noteworthy that *E*_vdw_ was about threefold higher than the Δ*E*_ele_, except in the case of apigenin complexed with the 5HT2AR. The current findings supply quantitative data on the binding affinities of apigenin and safranal as prospective 5HT2AR and 5HT1AR ligands, respectively.

### 2.3. Analysis of the Key Amino Acids Involved in Drug–Protein Interactions

**Figure 6:**
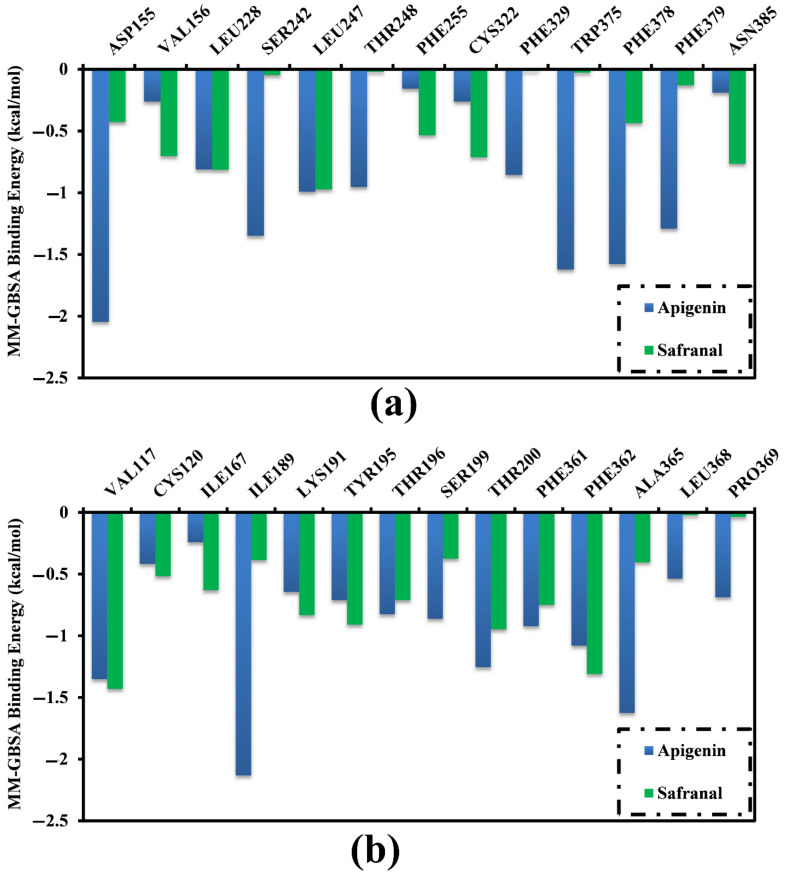
Per residue decomposition of the total binding affinity (kcal/mol) of apigenin and safranal complexed with (**a**) 5HT2AR and (**b**) 5HT1AR.

To identify the key amino acid residues of the 5HT2AR and 5HT1AR that were primarily involved in the interaction with apigenin and safranal, per residue decomposition of the binding affinity was carried out. All the amino acid residues with energetic participation <−0.50 kcal/mol were selected (Figure 6). The VAL156, LEU228, and LEU247 amino acid residues were the common residues of the 5HT2AR that interacted with both apigenin and safranal (Figure 6a). Similarly, the residues VAL117, ILE189, THR200, PHE 361, and PHE 362 were the common residues of the 5HT1AR that interacted with both apigenin and safranal (Figure 6b).

### 2.4. Post-Molecular Dynamics Analyses

While the docking and MD simulations combined with binding affinity estimations unveiled apigenin and safranal as prospective 5HT2AR and 5HT1AR ligands, respectively, further MD-based analyses were carried out to investigate the structural and energetic stabilizations for inhibitor-receptor complexes.

#### 2.4.1. Binding Affinity per Frame

**Figure 7:**
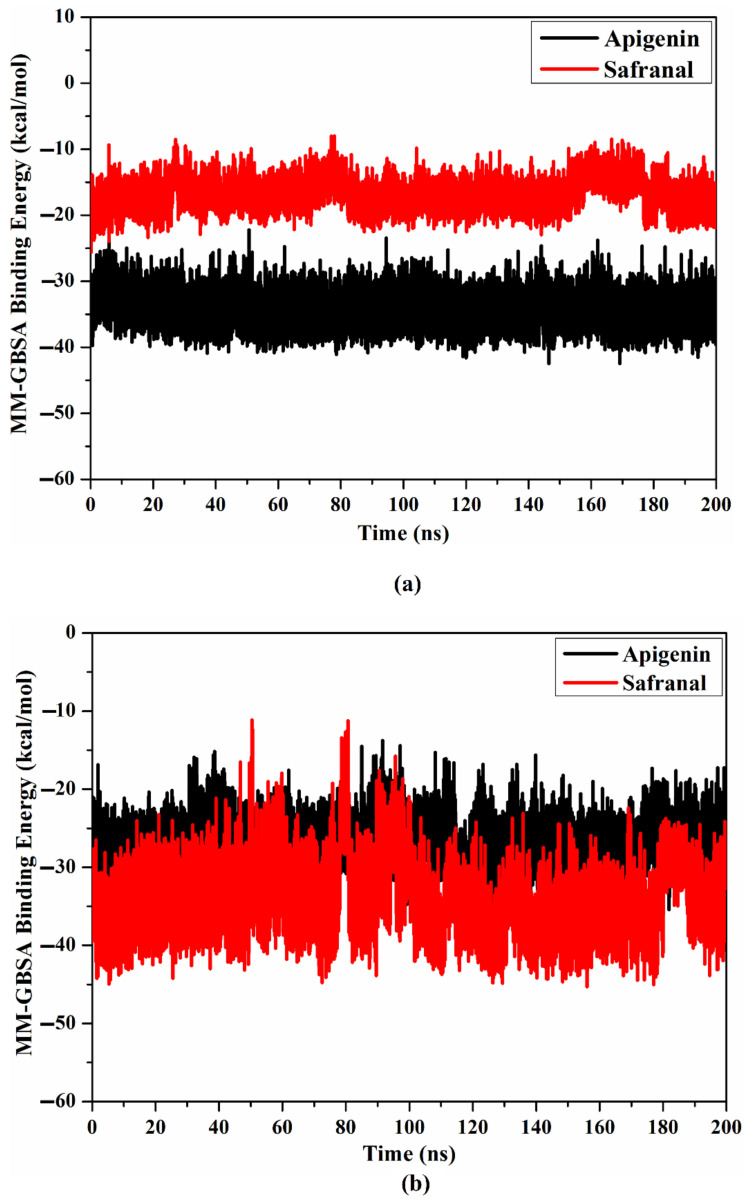
Evaluated binding affinity per frame for apigenin (in black) and safranal (in red) with the (**a**) 5HT2AR and (**b**) 5HT1AR throughout 200 ns MD simulations.

To measure the steadiness of apigenin and safranal within the binding site of the 5HT2AR and 5HT1AR, the correlation between the time and binding affinity was examined over the 200 ns MD simulations (Figure 7). As shown in Figure 7, the most exciting portion of the data was the comprehensive constancy of apigenin and safranal complexed with the 5HT2AR and 5HT1AR throughout 200 ns MD, with average binding energies of −34.4, and −17.1 as well as −26.8, and −22.3, respectively.

#### 2.4.2. Center of Mass Distance

**Figure 8:**
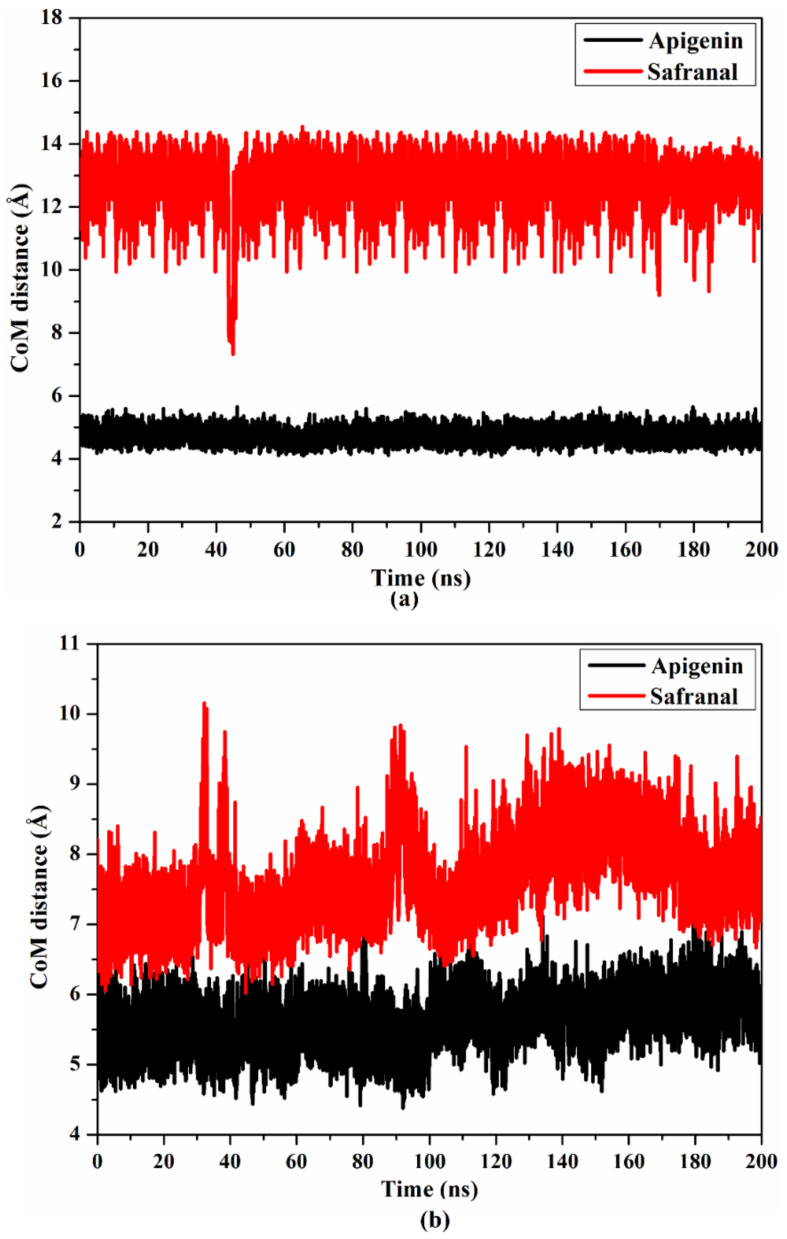
CoM distances (in Å) between apigenin and safranal and (**a**) VAL156 of the 5HT2AR as well as (**b**) VAL117 of the 5HT1AR during 200 ns MD simulations.

The center of mass (CoM) distances were calculated to warrant a wider perspective into the constancy of the investigated ligands complexed with the 5HT2AR and 5HT1AR throughout 200 ns MD (Figure 8). The CoM distances were found to be more consistent for apigenin than safranal in a complex with the 5HT2AR and 5HT1AR, with average values of 4.7 and 12.3 as well as 5.6 and 7.6 Å, respectively. The present data propose that apigenin binds more tightly to the 5HT2AR and 5HT1AR than safranal.

#### 2.4.3. Root Mean Square Deviation

**Figure 9:**
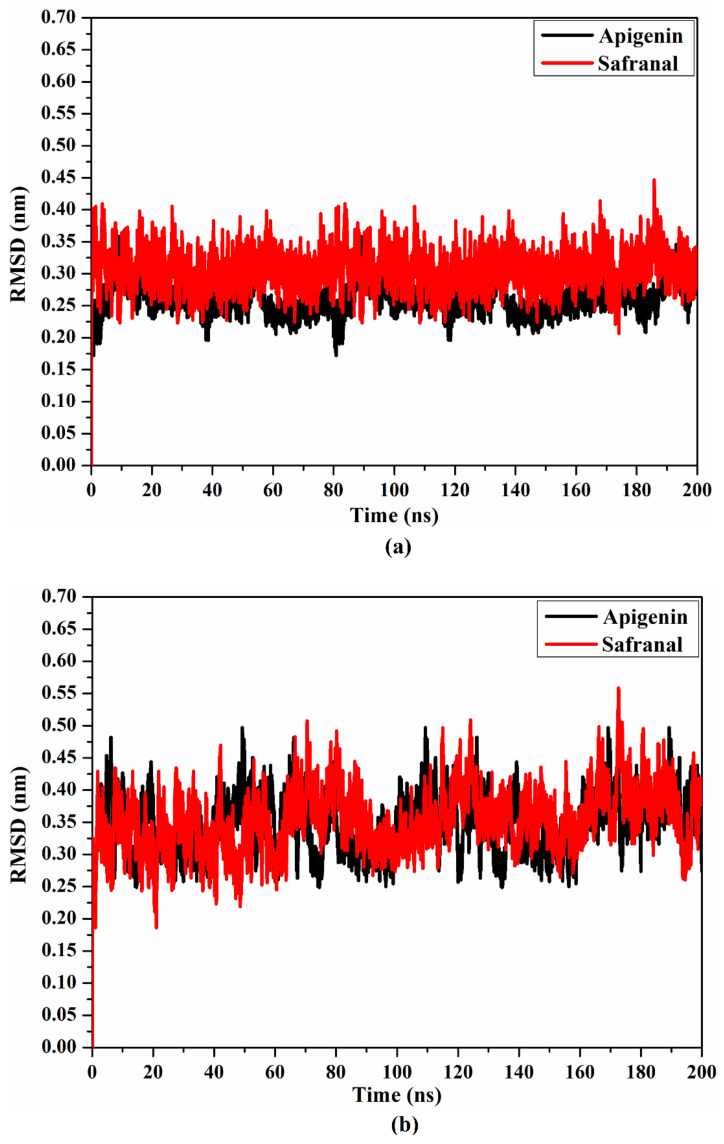
RMSD of apigenin (black) and safranal (red) with the (**a**) 5HT2AR and (**b**) 5HT1AR throughout the 200 ns MD simulations.

To inspect the conformational changes in the drug–5HT2AR and drug–5HT1AR complexes throughout the MD simulations, the root mean square deviation (RMSD) for the examined complexes was estimated. For apigenin and safranal in complexes with the 5HT2AR and 5HT1AR, the RMSD was noticed to be beneath 0.5 nm (Figure 9). Overall, the data suggest that apigenin tightly interacts with 5HT2AR and 5HT1AR, and does not influence the conformational constancy of the investigated receptors.

#### 2.4.4. Protein-Lipid System MD Simulations

**Figure 10:**
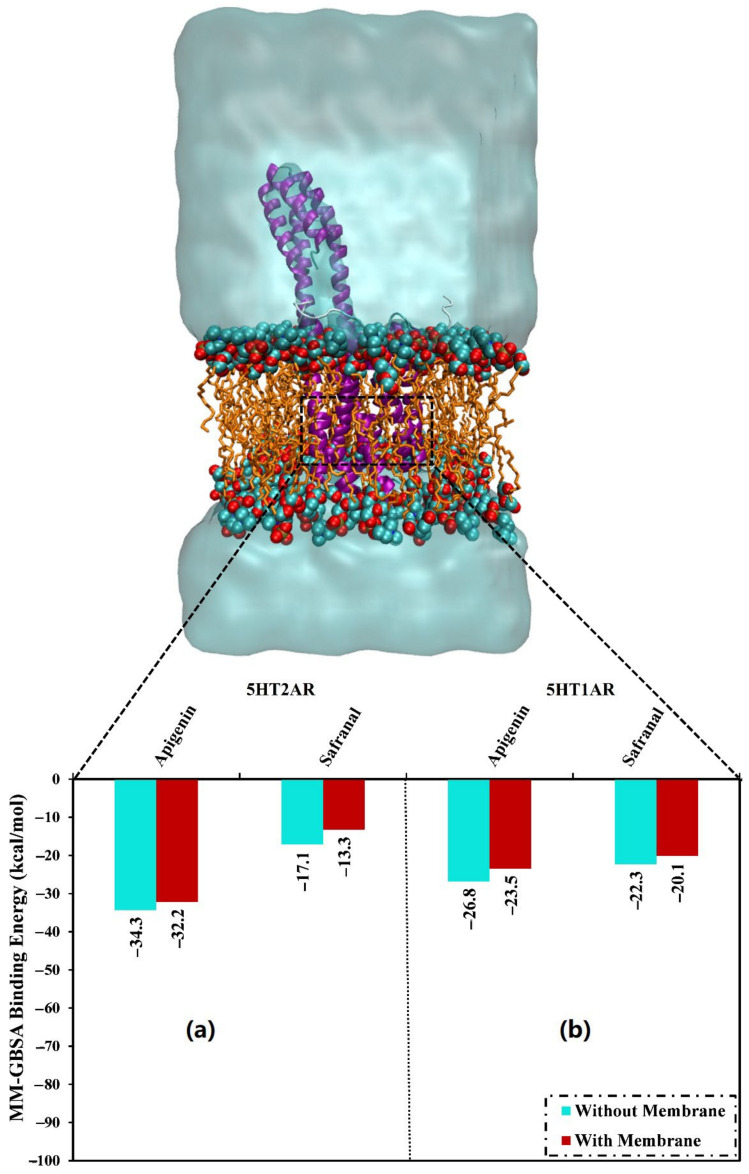
Binding affinities of the inspected apigenin and safranal with the (**a**) 5HT2AR and (**b**) 5HT1AR in the absence and presence of the POPC bilayer membrane over 200 ns MD simulation.

The presence of a membrane lipid bilayer in the MD simulations has a very considerable role that may affect the stability of the drug-receptor binding affinities. Thus, in the current study, the investigated inhibitors were simulated in the presence of a POPC membrane for 200 ns, and the binding energies were then estimated. In the presence of POPC, apigenin and safranal demonstrated binding energies (Δ*G*_binding_) of −32.2 and −13.3 as well as −23.5 and −20.1 against the 5HT2AR and 5HT1AR, respectively (Figure 10). The results of the binding energies in the presence and absence of a lipid membrane revealed that the presence of the lipid mediation slightly increased the ligand-receptor binding energy. For instance, apigenin complexed with the 5HT2AR exhibited binding energies (Δ*G*_binding_) of −32.2 and –34.3 kcal/mol in the presence and absence of a lipid membrane, respectively (Figure 10).

### 2.5. Drug-Likeness Evaluation

To assess the physicochemical properties, the drug-likeness parameters were predicted. The predicted physicochemical parameters are summarized in Table 2. The analysis showed the presence of permeability through the cell membrane within the measurement of the miLog*P* ≤ 5. The calculated miLog*P* values of apigenin and safranal were 2.5 and 3.0, respectively, suggesting that these ligands had good membrane permeability. To evaluate the absorption and membrane permeability profiles [44], the TPSA values of the investigated ligands were calculated. The results demonstrated that apigenin and safranal had average TPSA values of 90.9 and 17.1 Å^2^, respectively, proposing their absorption through the intestine (<140 Å^2^).

The molecular weights of the ligands were found to be less than 500 Da (calc. 270.2 and 150.2 Da for apigenin and safranal, respectively), suggesting that these inhibitors would be facilely transported, diffused, and absorbed. Finally, the number of hydrogen bond acceptors (nON) was five and one, and the number of hydrogen bond donors (nOHNH) was less than five for these investigated inhibitors.

### 2.6. EPMT and FST for Analyzing Behavioral Effects of Apigenin and Safranal in the Rodent Model of Depression

The influences of apigenin and safranal were measured after three weeks of treatment, using the FST and the EPMT as measures of their antidepressant and antianxiety actions, respectively.

**Figure 11:**
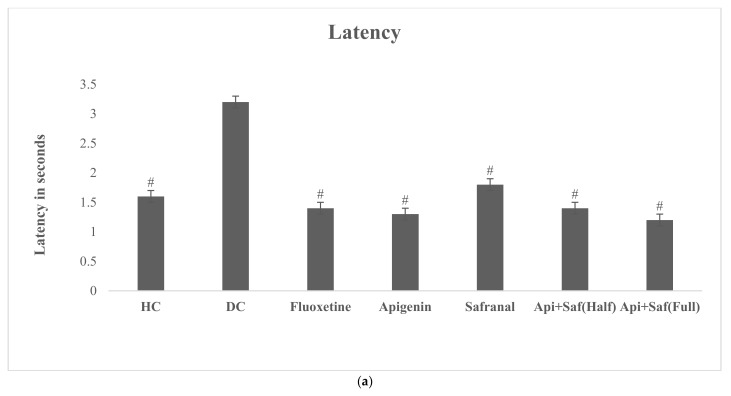

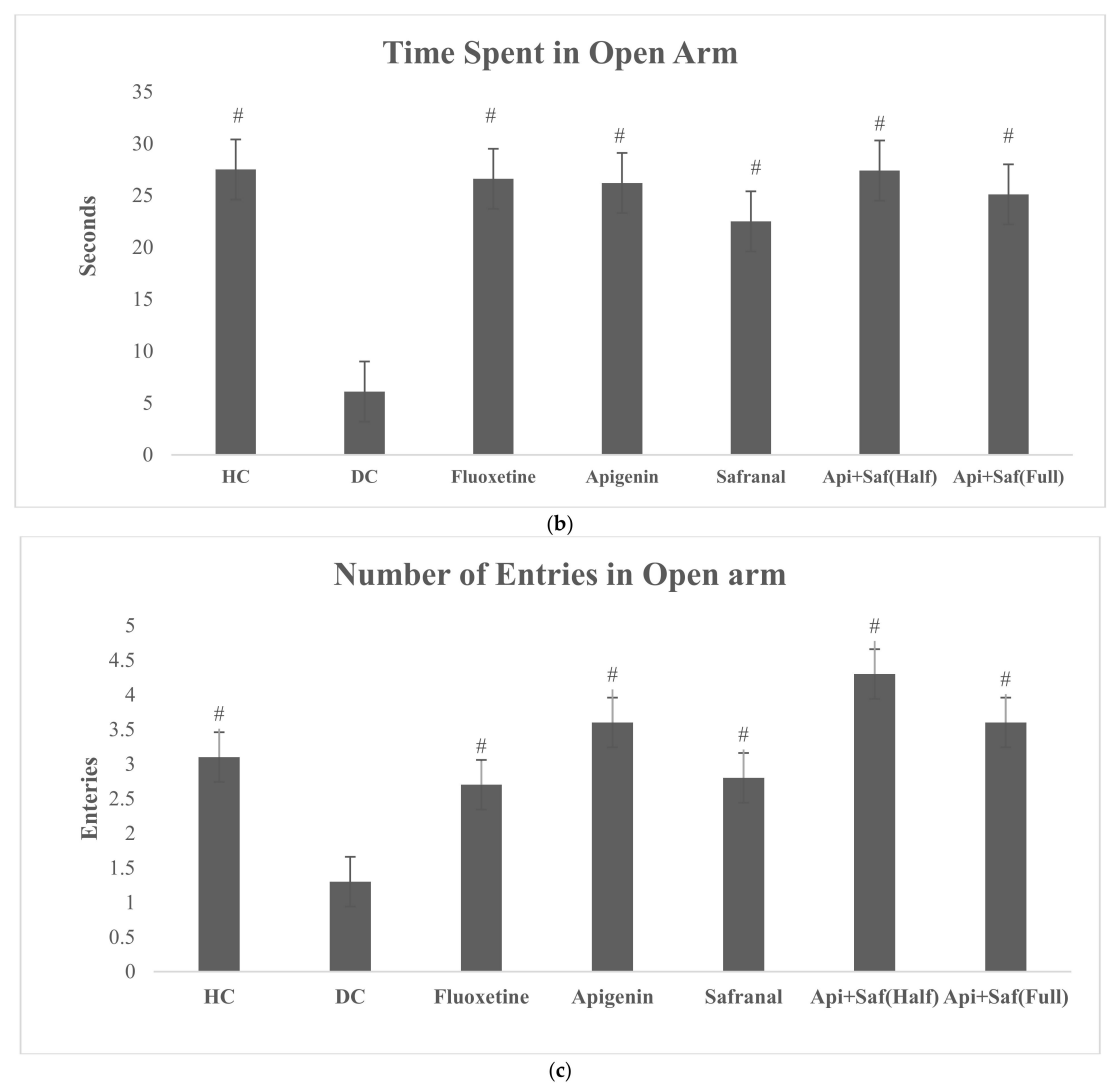
(**a**) Effects of three weeks of oral gavage of safranal, apigenin, and its combination on latency for EPMT in diabetic (induced) depressed rats. Values are shown as average ± SD (n = 10). One-way ANOVA was employed to analyze the results pursued by Tukey’s post hoc test. Here, # indicates significance (*p* < 0.05) compared with the diabetic control (DC). Key: healthy control (HC), diabetic control (DC), apigenin (Api), and safranal (Saf). (**b**) Effects of three weeks of oral gavage of safranal, apigenin, and its combination on time spent in open arm for EPMT in diabetic (induced) depressed rats. Values are shown as average ± SD (*n* = 10). One-way ANOVA was used to analyze the results, pursued by Tukey’s post hoc test. Here, # indicates significance (*p* < 0.05) compared with diabetic control (DC). Key: healthy control (HC), diabetic control (DC), apigenin (Api), and safranal (Saf). (**c**) Effects of three weeks of oral gavage of safranal, apigenin, and its combination on the number of entries in the open arm for EPMT in diabetic (induced) depressed rats. Values are shown as average ± SD (n = 10). One-way ANOVA was used to analyze the results, followed by Tukey’s post hoc test. Here, # indicates significance (*p* < 0.05) compared with diabetic control (DC). Key: healthy control (HC), diabetic control (DC), apigenin (Api), and safranal (Saf).

The analysis of the EPMT revealed a significant difference in latency, time spent in the open arm, and the number of entries in the open arm of treatment groups. The healthy control revealed a significantly lower (F_6.63_ = 7.82, *p* < 0.05) latency time, which was also seen in all treated groups, compared with the DC group (Figure 11a).

The time spent in the open arms (Figure 11b) was also determined, and increased activity (F_6.63_ = 29.915, *p* < 0.05) was monitored in all handled groups other than the DC group. The number of entries in the open arm (Figure 11c) was remarkably greater (F_6.63_ = 6.804, *p* < 0.05) in the apigenin, safranal, and half-dose combination treatment groups compared with the DC group. Moreover, a significantly higher (*p* < 0.05) number of entries was seen in the half-dose combination-treated group compared with the fluoxetine-treated group. However, no notable variation in any of the EPMT parameters was noticed between the half- and full-dose combination treatment groups.

**Figure 12:**
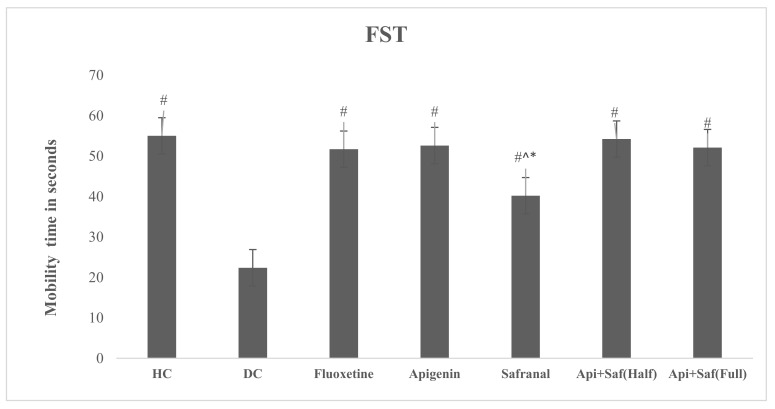
Effects of three weeks of oral (gavage) administration of safranal, apigenin, and its combination on FST in diabetic (induced) depressed rats. Values are shown as average ± SD (n = 10). One-way ANOVA was employed to analyze the results pursued by Tukey’s post hoc test. Here, # indicates significance (*p* < 0.05) compared with diabetic control (DC), * indicates significance (*p* < 0.05) compared with safranal and apigenin (half dose), and ^ indicates significance (*p* < 0.05) compared with safranal and apigenin (full dose). Key: healthy control (HC), diabetic control (DC), apigenin (Api), and safranal (Saf).

The FST analysis (Figure 12) showed a considerable rise in mobility time in all treated groups (*p* < 0.05) compared with the DC group. However, there was a significantly lower (F_6.63_ = 60.023, *p* < 0.05) mobility time in the safranal-treated group when compared with the HC and fluoxetine groups. Additionally, the safranal-treated group manifested remarkably diminished (*p* < 0.05) mobility compared with the full- and half-dose combination treatments of apigenin and safranal. The results showed an insignificant difference between the HC and fluoxetine-treated rats. Overall, the test groups, especially the full combination groups, showed the highest mobility periods and hence antidepressant effects compared with all the other groups in the study.

## 3. Discussion

This study was built on previously published observations of the in vivo efficiency of apigenin and safranal in the remediation of gloom [26,45,46]. Apigenin (4′,5,7-trihydroxyflavone) is present in chamomile as a part of the flavonoid component of this herb and functions to protect and heal the endocrine pancreas [47] and reduce the symptoms of depression [48]. These are important functions of apigenin, as there is a bidirectional relationship between these two diseases [49], making apigenin a potential antidepressant that is especially useful in pancreatic disorders such as diabetes mellitus, where depression and anxiety manifest as comorbid conditions [25]. Furthermore, apigenin has a well-established safety profile [50]. Similar to apigenin, safranal (2,6,6-Trimethyl-1,3-cyclohexadiene-1-carboxaldehyde) has effects on diabetes mellitus (DM) [51], major depressive disorder (MDD) [52] and anxiety [53]. The well-established safety profile of safranal [54,55] has been exploited, and studies have been conducted on its effectiveness against depression and anxiety in doses considered safe [56,57,58].

In silico approaches were utilized to predict the molecular interactions between safranal and apigenin and the 5HT1A and 5HT2A receptors, which may provide insights into the putative target sites of these natural agents within the two receptors. The in silico analyses proposed that apigenin and safranal can interact steadily with two of the essential serotonin receptors implicated in depression and anxiety, namely the 5HT2AR and 5HT1AR in their putative ligand-binding sites.

The docking analyses suggest a considerable overlap in the amino acid residues in the 5HT2AR and 5HT1AR targeted by both apigenin and safranal. Amino acids VAL156, LEU228, and LEU247 were the common residues of the 5HT2AR that interacted with both apigenin and safranal. The amino acid VAL156 is in TMH3, where it is a putative ligand-binding site (NCBI’s CDD (Conserved Domain Database)), while LEU228 and LEU247 are located in a transition loop between TMH4 and TMH5 and TMH5, respectively. Similarly, residues VAL117, ILE189, THR200, PHE361, and PHE362 were the common residues of the 5HT1AR that interacted with both apigenin and safranal. The amino acid VAL117 was mapped as part of TMH3 in a putative ligand-binding site (CDD), and ILE189 was located in the binding pocket between TMH4 and TMH5 [59], while THR200 and PHE361 and PHE362 were located on TMH5 and TMH6, respectively, where the latter two residues (PHE361 and PHE362) were in putative ligand-binding sites (CDD). These results suggest that these two molecules, apigenin, and safranal interact with similar residues in the 5HT2AR and 5HT1AR, where they might exert their functions at the level of these serotonin receptors.

MD simulation and MM-GBSA analyses suggested that both apigenin and safranal exhibited strong MM-GBSA binding energies in the putative ligand-binding sites of the 5HT2AR and 5HT1AR, being −34.3 and −17.1 kcal/mol as well as −26.8 and −22.3 kcal/mol, respectively, over 200 ns MD simulations (Table 1). VdW interactions were discovered to be the predominant factor involved in the formation of a molecular complex with the 5HT2AR and 5HT1AR for both apigenin and safranal. The van der Waals interactions became stronger and more relevant when the ligand-protein interaction was of a very close proximity, suggesting a good fit for the interaction [60]. Similar to this, the electrostatic force was also found to figure prominently into all of the interactions, as quantified in the results. Electrostatic interactions are key players in the determination of ligand-protein binding specificity as well as the rate of ligand-protein association [61]. This gives a strong guide for a possible function for apigenin and safranal in modulating the activity of the 5HT2AR and the 5HT1AR, which could serve to explain their effectiveness as antidepressants and anxiolytics. Post-MD analysis using the binding energy per frame further supported our hypothesis that there is a stable interaction of the ligands with the receptors (Figure 6). Apigenin was found to bind more consistently with both receptors, as indicated by the center of mass distances (Figure 7), in comparison with safranal. In terms of the RMSD, apigenin was found to preserve the structural integrity without compromising the structural stability of the two receptors.

The drug-likeness evaluation led us to conclude that these molecules have good permeability and absorption. Researchers have reported that apigenin affects the CNS directly [45,62], as measured in behavioral and histochemical assays on animal brains that have similar anatomies to humans. This indicates that apigenin crosses the BBB [60] and may be transported via transporters or passive diffusion [63].

The FST and EPMT are commonly used to screen drugs for antidepressant [64] and antianxiety [65] effects. In FST, the period of solidity reflects a situation of hopelessness, which is a key symptom of depression, whereas in EPMT, increased latency time, decreased time spent in the open arm, and a decreased number of entries in the open arm suggest a condition of anxiety [66]. STZ-induced diabetes in rodents increased the duration of latency, decreased the time spent in the open arm, and reduced the number of entries in the open arm in the EPMT, which is similar to other studies’ findings [67], while decreasing the mobility time in the FST [68]. The current results demonstrate the onset of depressive and anxiety-related symptoms resulting from altered brain monoamine levels and functions in diabetes. Apigenin and safranal therapy substantially reversed diabetes-induced altered behavior in FST and EPMT, and the impact was comparable to that of an antidepressant. The findings were consistent with prior research, indicating that apigenin and safranal have an antidepressant and anxiolytic effect in STZ-induced diabetes in rodents [69,70,71]. However, it is important to note that in the current study, we observed that the combination of the compounds as a half dose had a similar effect to that observed for full doses and when used alone or in combination. This effect may allow the use of lower doses for treatment, with further reduced side effects and costs associated with the use of apigenin and safranal. Notably, numerous studies have linked brain monoamine alterations to the despair effects in the FST and EPMT models [72,73]. The increased mobility time in the FST and more time spent in the open arms in the EPMT are linked to increased monoamines and, more specifically, serotonin activity in the brain [74]. As a result, the possible mechanism underlying the effects of apigenin and safranal may involve monoaminergic modulations, especially serotonin.

The limitation of the current study is that no in vitro experiments to determine receptor inhibition were performed, due to the unavailability of experimental facilities in our laboratory. However, we believe that the in vivo arm of this study helped us investigate the actual effects that apigenin and safranal have on mood symptoms, thus validating the efficacy of these compounds in ameliorating MDD and anxiety in an established murine model. The murine model used in this study is well-established [39], and many studies have employed streptozotocin to render this model of mood disorders [28,29,30,31]. Therefore, we found evidence of a validated interaction of apigenin and safranal with the 5HT1A and 5HT2A receptors, which supports the direct involvement of serotonin in the antidepressant and anxiolytic effects of these compounds.

## 4. Materials and Methods

### 4.1. Molecular Structures of Safranal and Apigenin and the Protein Structures of the 5HT2AR and 5HT1AR

The structures of apigenin and safranal were obtained from PubChem [75] in SDF format. Using Open Babel [76] software, the SDF files were converted to pdbqt format. The 3D structures of the 5HT2AR and 5HT1AR were retrieved from RCSB PDB (PDB codes: 6a93 [42] and 7e2z [43], respectively). The experimental resolution of the selected PDB structures was 3.00 Å. The protein structures were prepared by stripping out all ions, crystallographic waters, heteroatoms, and bound ligands, preserving only the amino acid residues of the 5HT2AR and 5HT1AR. All missing amino acids were constructed utilizing MODELLER software [77]. The protonation states of the amino acids of the studied receptors were investigated by the H++ web server [78]. Aside from that, the missing hydrogen atoms were added [78]. The following physical conditions were considered in the protonation state estimations: external dielectric = 80, salinity = 0.15, pH = 6.5, and internal dielectric = 10.

### 4.2. Molecular Docking

The PDB structures of the 5HT1A and 5HT2A receptors were saved in pdbqt format utilizing AutoDock Tools [79]. The AutoDock protocol [80] was followed during the pdbqt file preparation. AutoDock Vina1.1.2 software was used to perform all docking computations [81]. For the investigated receptors, a grid with a box size of 20 Å × 20 Å × 20 Å was employed. A grid spacing value of 1.0 Å was utilized in the docking computations. The exhaustiveness value was set at 200 for all docking calculations. BIOVIA Discovery Studio Visualize 2020 was utilized to visualize the docking poses and to analyze the ligand-receptor interactions [82].

### 4.3. Molecular Dynamics

Molecular dynamics (MD) simulations for the investigated ligands complexed with the 5HT1A and 5HT2A receptors were executed utilizing AMBER16 software [83]. AMBER force field 14SB [84] and a general AMBER force field (GAFF2) [85] were employed to characterize the receptors and ligands, respectively. The restrained electrostatic potential (RESP) approach [86] was used to compute the charges of the investigated ligands at the HF/6-31G* level utilizing Gaussian09 software [87]. All investigated systems were subsequently water solvated with an average distance of 12 Å between the truncated octahedron box edge and the atoms of the scrutinized complexes. The solvated systems were minimized for 5000 steps. Subsequently, the minimized systems were heated and adequately equilibrated for 10 ns. The equilibrated ensembles were eventually submitted to MD simulations for 200 ns. During the MD simulations, the particle mesh Ewald (PME) method was utilized to handle the electrostatic conditions [88]. The time step of the MD simulations was adjusted to 0.002 ps. Additionally, a 12 Å cut-off was employed for nonbonded interactions. The temperature was conserved at 298 K by utilizing the Langevin dynamics with a collision frequency gamma_ln = 1.0 ps^−1^. The Berendsen barostat was employed to regulate the pressure, with a relaxation time of 2 ps [89]. All bonds involving hydrogen atoms were constrained by utilizing the SHAKE method [90]. The trajectories of the simulated complexes were saved every 10 ps for binding affinity calculations and post-dynamics analyses. The PMEMD.cuda engine implemented inside AMBER16 software was employed to perform all MD simulations.

#### 4.3.1. Protein-Lipid Complex Construction

A membrane patch made of 1-palmitoyl-2-oleoyl-phosphatidylcholine (POPC) was generated using the CHARMM-GUI web-based server for the investigated inhibitors against the 5HT1A and 5HT2A receptors [91]. The complexes were positioned with their long axes parallel to the lipid surface in the middle of the POPC lipid bilayer. The entire systems were solvated by employing TIP3P water molecules and neutralized using sodium (Na^+^) and chloride (Cl^−^) ions at a concentration of 0.15 M. Finally, using the prepared systems, MD simulations were performed by employing a previously described procedure with parametrizing of the POPC membrane using the Amber lipid14 force field.

#### 4.3.2. Binding Energy Computations

The binding affinities for the investigated ligands in complex with the 5HT1A and 5HT2A receptors were calculated by utilizing the molecular mechanically generalized Born surface area (MM-GBSA) method [92]. The polar solvation energy was assigned, utilizing the adjusted GB model developed by Onufriev (igb = 2) [93]. The snapshots were recorded every 10 ps over the production stage of a 200 ns MD simulation, giving a total of 20,000 snapshots. The MM-GBSA binding energies were estimated based on the uncorrelated trajectories collected from the MD and lipid-mediated MD simulations.

### 4.4. Prediction of the Physicochemical Features of Apigenin and Safranal

Molinspiration (http://www.molinspiration.com; accessed 30 September 2021) was applied for estimating the physicochemical properties, such as the hydrogen bond acceptor (nON), molecular weight (MW), rotatable bond count (RB), octanol/water partition coefficient (miLog*P*), topological polar surface area (TPSA), and hydrogen bond donor (nOHNH). An OpenBabel server was utilized to convert the investigated ligands into SMILE format to analyze the molecular properties [76].

#### 4.4.1. In Vivo Rodent Model for Analysis of the Behavioral Effects of Apigenin and Safranal on Depression and Anxiety

Pure chemical compounds of safranal (Sigma-Aldrich, St. Louis, MO, USA; #116-26-7, with a purity of 90%) and apigenin (Ambeed, Arlington Heights, IL, USA; 5,7-Dihydroxy-2-(4-hydroxyphenyl)-4H-chromen-4-one #520-36-5, with a purity of 98%) components were purchased from Life Sciences Inc. and Techsource enterprises, respectively.

Seventy locally bred Sprague Dawley rats (weighing 200 g ± 20 g) were purchased from the Dow University of Health and Sciences in Karachi. All the rats were acclimatized for a week in a separate cage under a 12 h light-dark cycle and at a constant room temperature from 22 °C to 25 °C with a free excess of standard feed and water. This research was authorized by the ethical review committee of the Federal Urdu University of Arts, Science, and Technology in Karachi. The 70 animals were randomly divided into the following 7 groups (n = 10/group): (1) healthy control (HC) with normal rats, (2) disease control (DC) with diabetic rats, (3) fluoxetine-treated diabetic rats, (4) safranal-treated diabetic rats, (5) apigenin-treated diabetic rats, (6) a half safranal-apigenin combination treatment group, with a combination of safranal and apigenin as a half dose administered to diabetic rats, and (7) a full safranal-apigenin treatment group, with safranal and apigenin administered to diabetic rats as a full dose. Oral gavage was used to administer the drugs and active compounds once a day for three weeks. At the end of the experiment, behavioral analysis was conducted for the evaluation of anxiety and depression in the animals.

#### 4.4.2. Diabetes Induction in Rats

Streptozotocin (STZ) was freshly synthesized in a citrate buffer (0.1 M, pH 4.5) for intraperitoneal injection at a dose of 60 mg/kg to induce diabetes in disease control (DC) fluoxetine-treated diabetic rats and diabetic test rats (administered with active compounds alone or in combination) [94,95].

#### 4.4.3. Administration of Drugs

Fluoxetine was prepared in saline and administered by oral gavage at a dosage of 5 mg/kg [96] for 3 weeks. Safranal was diluted in 10% Kolliphor EL (Sigma-Aldrich, St. Louis, MI, USA; #C5135) and given orally via gavage for 3 weeks at doses of 0.5 mg/kg using a gavage needle [97]. Apigenin was dissolved at a concentration of 10 mg/mL in distilled water with 5% sodium carboxyl methylcellulose (CMC-Na) and given orally for 3 weeks at a dose of 50 mg/kg [98]. The combination of the full dose was used as 50 mg/kg of apigenin with 0.5 mg/kg of safranal. At the same time, the half dose was prepared as 25 mg/kg of apigenin and 0.25 mg/kg of safranal.

#### 4.4.4. Elevated Plus Maze Test (EPMT)

The elevated plus maze test (EPMT) was utilized to study anxiety in rats [65]. The experiment employed a maze with four equally sized arms, with two open arms crossed by two closed arms. The closed arms were bounded by a 40 cm wall on three sides. These arms were connected by a central square (10 × 10 cm^2^) that gave the maze a plus sign appearance. The EPMT was 60 cm above the ground. Each rat was placed at the end of the open arm such that it faced the opposite direction of the center square. For 5 min, the latency, time spent in the closed arm, and time spent in the open arm were recorded [99].

#### 4.4.5. Forced Swim Test (FST)

The forced swim test (FST) is used to determine depression in rodents [64]. The dimensions of the FST apparatus were 56 cm × 30 cm (h × w). The container was filled with water up to a height of 22 cm at a temperature of 25 °C. The water depth was adjusted to prevent the tail from coming in contact with the bottom of the container and to avoid the animals escaping from the apparatus. The animals were allowed to swim for 5 min, and their total mobility time was recorded in seconds [100].

### 4.5. Statistical Analysis

The mean values obtained for each group were statistically analyzed using one-way ANOVA in SPSS version 22.0, followed by a post hoc Tukey’s test. Alpha values of *p* < 0.05 were considered significant.

## 5. Conclusions

In conclusion, the in silico and in vivo analyses indicated that there is an antidepressant and anxiolytic effect of individual apigenin and safranal and their combination, which can be linked to their docking effectively on the serotonin receptors (5HT2A and 5HT1A) in particular. Since NCBI (conserved domain database) showed that the amino acids in the binding pockets in the docking interactions of the two ligands with the respective receptors were mostly in the putative ligand-binding sites for these receptors, we can hypothesize that the antidepressant and anxiolytic activity of these ligands would follow predictable patterns, which include possible inhibition of the 5HT2AR and agonism of the 5HT1AR. The immersion of the apigenin and safranal complexed with the serotonin receptors (5HT2A and 5HT1A) in a POPC bilayer demonstrated no significant impact on the inhibitor’s binding affinity. Overall, our results show that the interactions of apigenin and safranal with the serotonin receptors, as evident from the in silico evaluation, may help alleviate depression and anxiety, as demonstrated in the behavioral assays performed in a standard model of the anxio-depressive states in rats. The anxio-depressive DM model was based on earlier research by other investigators [39] and has been used widely by others since then [28,29,30,31]. While it is a strength of this study that we combined an in silico method with in vivo behavioral tests, our laboratory is not yet equipped to perform a suitable bridging in vitro investigation, such as X-ray crystallography, NMR, or an ITC. This can then be a future direction of the current evaluation of apigenin and safranal as antidepressants and anxiolytics.

## Figures and Tables

**Table 1:** Calculated docking scores, binding features, and binding affinities during a 200 ns MD simulation of apigenin and safranal against 5HT1AR and 5HT2AR.

Receptor	Compound Name	Docking Score (kcal/mol)	Binding Features ^a,b^	MM-GBSA Binding Energy (kcal/mol)
5HT2A	Apigenin	−8.9	ASP155 (1.69 Å)	−34.3
			LEU228 (2.32 Å)	
			SER242 (2.45 Å)	
	Safranal	−6.8	ASN385 (1.90 Å)	−17.1
5HT1A	Apigenin	−8.4	THR121 (2.57 Å)	−26.8
			THR196 (1.88 Å)	
	Safranal	−6.0	----- ^c^	−22.3

^a^ Inspected based on the last trajectory of the 200 ns MD simulations. ^b^ Only hydrogen bonds (in Å) were demonstrated. ^c^ No hydrogen bond was observed.

**Table 2:** Anticipated physiochemical characteristics of apigenin and safranal as putative 5HT2AR and 5HT1AR inhibitors and agonists, respectively.

Compound Name	miLog*P*	TPSA	nON	nOHNH	Nrotb	MVol	MWt	%ABS
Apigenin	2.5	90.9	5	3	1	224.1	270.2	77.6%
Safranal	3.0	17.1	1	0	1	158.6	150.2	103.1%

## Data Availability

Not applicable.

## Sample Availability

Samples of the compounds (apigenin and safranal) are available from the authors.
